# ABO Blood Group and Rhesus (Rh) Antigen Distribution Among Blood Donors in the North Al Sharqiyah Governorate in Oman

**DOI:** 10.7759/cureus.94669

**Published:** 2025-10-15

**Authors:** Musleh Al Musalhi, Abdulrahman Al Saifi, Khalid Al Matari, Ibrahim Al Habsi

**Affiliations:** 1 Hematopathology, Ibra Hospital, Ibra, OMN

**Keywords:** abo blood group system, alloimmunization, blood donors, oman, phenotype, rhesus (rh) antigens

## Abstract

Background

The distribution of ABO and Rhesus (Rh) blood group antigens varies across different geographical and ethnic populations. Understanding these indigenous variations is crucial for effective blood bank management, ensuring safe transfusion practices, and preventing alloimmunization, particularly in multi-transfused cases. While national data for Oman exists, specific data for the North Al Sharqiyah Governorate has been lacking. This study aimed to determine the distribution of ABO and the five major Rh antigens (D, C, c, E, e) among volunteer blood donors in the North Al Sharqiyah Governorate of Oman and to compare these frequencies with established national data to identify significant local variations.

Methods

This is a retrospective, cross-sectional study conducted at the laboratory department in Ibra Hospital over a 35-month period (December 2017 - October 2020). Data from 1,433 eligible volunteer blood donors were analyzed. ABO and RhD typing, along with Rh phenotyping for C, c, E, and e antigens, were performed using standardized serological gel card techniques. Frequencies were calculated and compared to published national Omani data using the chi-squared test.

Results

The most common blood group was O positive (55.1%), followed by A positive (26.6%). Overall, 88.1% of donors were RhD positive and 11.9% were RhD negative. The most prevalent Rh antigen was 'e' (97.1%), and the least common was 'E' (19.9%). Significant regional differences were observed when compared to national data: a higher frequency of group A (29.3% vs. 18%; chi-square= 17.605, *p* value < 0.0001) and a lower frequency of group B (6.2% vs. 22.9%; chi-square= 89.47,* p* value < 0.0001). Notably, the R1R2 (DCe/DcE) phenotype was significantly less common in our cohort (9.0%) compared to the general Omani population (18.3%; chi-square = 24.54, *p* value < 0.0001). The R^0^r (Dce/ce) phenotype prevalence is higher among study donors than the Oman population (9.07 vs. 5.7; chi-square = 4.012, *p*-value = 0.0452).

Conclusion

This study reveals a distinct ABO and Rh phenotype distribution among blood donors in the North Al Sharqiyah Governorate compared to the national profile. The findings highlight a regional need for targeted blood donor recruitment, particularly for group B, and underscore the importance of local epidemiological data for optimizing blood inventory and enhancing transfusion safety.

## Introduction

The ABO and Rhesus (Rh) blood group systems are the most clinically significant in the blood transfusion field. The antigens of these systems are highly immunogenic, and incompatibility between a blood donor and a recipient can lead to severe, sometimes fatal, hemolytic transfusion reactions [1]. The repeated transfusions of phenotypically mismatched blood can lead to alloimmunization. This complication is particularly prevalent in chronically transfused patients, such as those with thalassemia or sickle cell disease, making subsequent transfusions more complex and hazardous. The prevalence of alloimmunization can reach as high as 75 % [2-4].

The distribution of ABO and Rh blood groups is known to vary significantly among different ethnic groups and geographic populations worldwide [5-7]. For instance, blood group O is the most common group globally, but its frequency is higher in populations from the Americas and Western Europe [8]. Group type A is common in central and eastern Europe and can reach a percentage of 45-50% of the population. In Asia, group B is more prevalent in Chinese and Indians [8]. Similarly, the Rh-negative phenotype is more common in Caucasian populations (15%) than in African (8%) or Asian populations (<1%). The DCe Rh haplotype is most frequently seen among individuals of Caucasian, Asian, and Native American descent, while in Black populations, the Dce haplotype tends to be slightly more prevalent [9].

Knowledge of local and regional blood group antigen frequencies is fundamental for effective blood bank management. It enables the forecasting of blood supply needs, the implementation of efficient donor recruitment strategies, and the maintenance of an adequate and balanced inventory of different blood types [10]. Furthermore, for multi-transfused patients, providing Rh- and Kell-phenotyped matched blood is a recommended strategy to prevent alloimmunization [11]. This requires a detailed understanding of the antigen phenotype prevalence within the local donor pool.

In the Sultanate of Oman, a study by Al-Riyami et al. established a national profile of blood group frequencies and phenotypes [12]. However, regional variations within a country can exist due to historical migration patterns and tribal distributions. The North Al Sharqiyah Governorate is a distinct region in Oman, and data specific to its population are essential for the local healthcare system.

Therefore, the primary aim of this study was to determine the distribution of ABO and the five major Rh antigens (D, C, c, E, e) among volunteer blood donors in the North Al Sharqiyah Governorate. A secondary aim was to compare Rh phenotype frequencies with the established national data to identify any significant local variations that could impact transfusion practices.

## Materials and methods

Study design and setting

This is a retrospective, cross-sectional study. It was conducted at the laboratory department of Ibra Hospital, a major secondary care hospital in the North Al Sharqiyah Governorate of the Sultanate of Oman. Data were collected from volunteer blood donors over a 35-month period from December 2017 to October 2020. Ethical approval was obtained from the Research and Studies Committee in the Directorate General of Health Services at the Ministry of Health (MoH/CSR/25/30466).

Study population

The study population consisted of healthy, unpaid volunteer blood donors who successfully donated blood during the study period. Donors were required to meet the eligibility criteria of blood donation set by the Omani Ministry of Health's Department of Blood Services. Donors with incomplete records or those who were deferred for positive transfusion-transmitted infection or not fitting the donation criteria were excluded from the final analysis. Also, repeated donations and records were excluded.

Data and sample collection

For each donor, demographic data (age, sex) and donation history were recorded. A blood sample was collected in an ethylenediaminetetraacetic acid anticoagulant tube for serological testing, with a minimal volume of 4 mL. The samples were tested within 24 hours of collection.

Serological testing

The ABO group and RhD status of donor samples were determined using a standardized serological gel column agglutination methodology. Comprehensive immunohematological evaluation, including both forward (red cell) and reverse (serum) typing, was performed with Bio-Rad© DiaClon ABO/D and Bio-Rad© DiaClon ABD-confirmation for donors ID cards (DiaMed GmbH, Cressier, Switzerland). Furthermore, Rh subgroup phenotype was accomplished serologically using Bio-Rad© DiaClon Rh-subgroups + Cw +K ID-cards containing monoclonal antisera (DiaMed GmbH, Cressier, Switzerland).

The standard operating procedure involved the preparation of a 5% red blood cell suspension by combining 25.0 µL of packed red cells with 0.5 mL of ID-Diluent 2 (DiaMed GmbH, Cressier, Switzerland). A calibrated pipette was used to dispense 12.5 µL of the cell suspension into the microtubes of the ID card. Following centrifugation in a dedicated ID-centrifuge for 10 minutes at 900 rpm, agglutination reactions were graded by visual inspection to determine the final phenotype.

Statistical analysis

Data were entered into a Microsoft Excel spreadsheet version 2509 (Microsoft Corp., Redmond, WA, USA). Frequencies and percentages were calculated for all ABO groups and Rh antigens. The results were tabulated and compared with the published national data from Al-Riyami et al. [12]. The chi-squared test was used to compare the percentage between Rh phenotype groups. Statistical significance was established at a threshold of p < 0.05, corresponding to a 95% confidence interval. The data were analyzed by using MedCalc® Statistical Software version 23.2.7 (MedCalc Software Ltd, Ostend, Belgium; 2025). 

## Results

A total of 1,433 eligible blood donors were included in the final analysis. The majority of donors were male (94.8%), with an age range of 18 to 64 years.

Distribution of ABO and RhD blood groups 

The distribution of ABO and RhD blood groups is presented in Table 1. The most common blood group was O positive (n=790, 55.1%), followed by A positive (n=381, 26.6%). The rarest groups were B negative (n=7, 0.5%) and AB positive (n=9, 0.6%). No donors with the AB negative blood group were found in this cohort. Overall, 88.1% of the donors were RhD positive, and 11.9% were RhD negative. 

**Table 1:** Frequency of ABO and RhD Blood Groups (n=1433)

Blood Group	Frequency (Percentage)	RhD status	Frequency (Percentage)
O	916 (63.9%)	Positive	790 (55.1%)
		Negative	126 (8.8%)
A	419 (29.3%)	Positive	381 (26.6%)
		Negative	38 (2.7%)
B	89 (6.2%)	Positive	82 (5.7%)
		Negative	7 (0.5%)
AB	9 (0.6%)	Positive	9 (0.6%)
		Negative	0 (0%)
Total	1433 (100%)		1433 (100%)

Frequency of Rh antigens among the study blood donors

**Figure 1:**
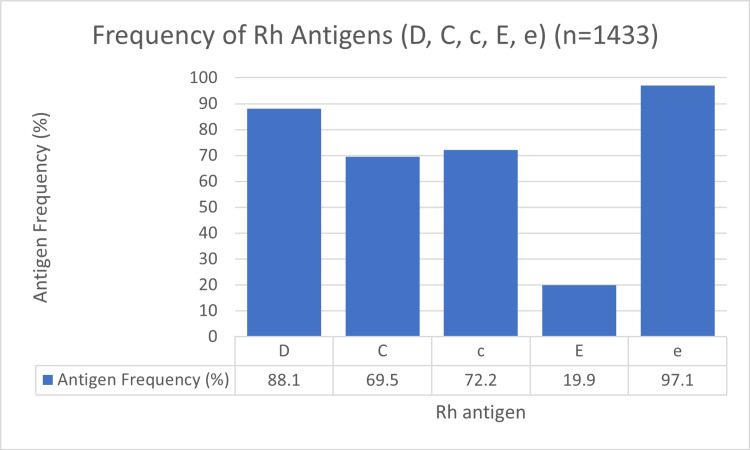
Frequency of Rh Antigens (D, C, c, E, e) (n=1433)

The frequencies of the five principal Rh antigens are detailed in Figure 1. The e antigen was the most prevalent, found in 97.1% of donors, while the E antigen was the least common at 19.9%.

Frequency of Rh phenotypes among the study blood donors

The distribution of the most common Rh phenotypes in our study cohort was compared to the national data published by Al-Riyami et al. [12] (Table 2). The most frequent phenotype in our population was R1r (DCe/ce) at 31.75%, which is comparable to the national figure. However, a notable difference was observed for the R1R2 (DCe/DcE) phenotype, which had a frequency of 9.0% in our study compared to 18.3% in the Omani population (chi-square = 24.54, DF=1, p-value < 0.0001). We observed that the R^0^r (Dce/ce) phenotype was higher than the Oman population with a significant difference of 9.07 vs. 5.7 (chi-square = 4.012, DF=1, p-value = 0.0452).

**Table 2:** Comparison of Rh Phenotypes in North Al Sharqiyah Donors vs. Study Donors in the Study by Al-Riyami et al. ¹Data adapted from Al-Riyami et al., 2019 [12] under Creative Commons Attribution-NonCommercial 4.0 International License. NA: non-applicable.

Rh Antigens					Frequency %		Test statistics		
D	C	c	E	e	Study Donors (n=1433) (%)	Donors^1^ from the 2019 Study by Al-Riyami et al. (n = 337) (%)	Chi-square	Degree of Freedom	p-value
+	+	-	-	+	27.77	22.8	3.431	1	0.0640
+	-	+	+	-	2.58	1.5	1.372	1	0.2414
+	-	+	-	+	9.07	5.7	4.012	1	0.0452
+	+	-	+	-	0.0	0.0	NA	NA	NA
+	+	+	-	+	31.75	32.6	0.0907	1	0.7633
+	-	+	+	+	7.68	8.1	0.0672	1	0.7955
+	+	-	+	+	0.0	0.0	NA	NA	NA
+	+	+	+	-	0.14	0.3	0.410	1	0.5219
+	+	+	+	+	9.0	18.3	24.540	1	< 0.0001
-	+	-	-	+	0.07	0.0	0.236	1	0.6272
-	-	+	+	-	0.07	0.0	0.236	1	0.6272
-	-	+	-	+	10.75	10.5	0.0178	1	0.8938
-	+	-	+	-	0.0	0.0	NA	NA	NA
-	+	+	-	+	0.63	0.3	0.527	1	0.4681
-	-	+	+	+	0.35	0.0	1.182	1	0.2769
-	+	-	+	+	0.0	0.0	NA	NA	NA
-	+	+	+	-	0.0	0.0	NA	NA	NA
-	+	+	+	+	0.07	0.0	0.236	1	0.6272

## Discussion

This study provides the first detailed report on the distribution of ABO and Rh blood group antigens among blood donors in the North Al Sharqiyah Governorate. Such regional data are vital for optimizing transfusion services and developing targeted public health strategies for recruiting blood donors.

The predominance of blood group O (63.9%) in our cohort is consistent with findings from across the Arabian Peninsula and globally [6,12-14]. However, when comparing our results with the national Omani data reported by Al-Riyami et al. [12], we observed a higher frequency of group A (29.3% vs. 18%, chi-squared= 17.605, p-value < 0.0001) and a lower frequency of group B (6.2% vs. 22.9%, chi-squared= 89.470, p-value < 0.0001). This regional variation could be attributed to the unique tribal and ancestral makeup of the population in North Al Sharqiyah. A higher prevalence of blood group A than group B was reported in different countries, such as Ethiopia [15], China [16], Somalia [17], and parts of Saudi Arabia [18]. This finding has direct implications for the Ibra Hospital blood bank, suggesting a need to focus donor recruitment efforts on individuals with group B blood to meet local patient needs adequately.

The frequency of RhD-negative individuals in our study was 11.9%, which is higher than the national average of 10.7% [12]. This is a critical finding for inventory management, as it highlights a substantial local need for RhD-negative blood units, which are often in short supply.

The analysis of Rh phenotypes revealed several important points. The high prevalence of the e antigen (97.1%) and the relatively low prevalence of the E antigen (19.9%) are typical for most populations [9]. The most significant finding from our phenotype analysis was the markedly lower frequency of the R1R2 (DCe/DcE) phenotype (9.0%) compared to the 18.3% reported for the general Omani population [12]. The R1R2 phenotype is clinically important because these individuals express all antigens. Transfusion of this phenotype can stimulate the production of alloantibodies if the patient has had negative antigen. Therefore, the less common of this phenotype can reduce the risk of alloimmunization [19].

The findings of this study reinforce the principle that blood group frequencies are not uniform, even within a single country. The observed differences between our regional data and the national data highlight the risk of relying solely on national-level statistics for local blood bank operations. This local data can be used to create a regional donor registry with extended phenotype information, which would be an invaluable resource for providing safe and compatible blood for chronically transfused patients, thereby reducing the risk of alloimmunization.

This study has some limitations. First, it was conducted at a single center, and while Ibra Hospital is a major hub, the findings may not be fully representative of the entire North Al Sharqiyah Governorate. Second, the study was limited to the ABO and Rh systems; data on other clinically significant blood groups, like Kell, Duffy, and Kidd, were not collected. The retrospective study design also introduces potential constraints, including incomplete data and selection bias. Furthermore, the comparative study referenced comprised a small sample size, thereby limiting the generalizability of its results to the national level. Future research should aim for a multi-center study and include a more extensive antigen typing profile.

## Conclusions

This study successfully characterized the distribution of ABO and Rh blood group antigens in a large cohort of blood donors from North Al Sharqiyah, Oman. We identified a regional pattern characterized by a higher prevalence of blood group A and R^0^r Rh phenotype and a lower prevalence of the R1R2 Rh phenotype compared to national figures. These findings are of significant practical value for the local blood transfusion service, aiding in the development of more effective donor recruitment campaigns and the optimization of blood inventory. We recommend that all regional blood banks undertake similar studies to ensure transfusion practices are guided by precise, local epidemiological data.
